# A Multiplex “Disposable Photonics” Biosensor Platform and Its Application to Antibody Profiling in Upper Respiratory Disease

**DOI:** 10.1021/acssensors.3c02225

**Published:** 2024-03-29

**Authors:** Michael R. Bryan, Jordan N. Butt, Zhong Ding, Natalya Tokranova, Nathaniel Cady, Brian Piorek, Carl Meinhart, Joshua Tice, Benjamin L. Miller

**Affiliations:** † Department of Dermatology, University of Rochester, Rochester, New York 14627, United States; ‡ Department of Biochemistry and Biophysics, University of Rochester, Rochester, New York 14627, United States; § Department of Chemistry, University of Rochester, Rochester, New York 14627, United States; ∥ Department of Nanoscale Science & Engineering, University at Albany, Albany, New York 12203, United States; ⊥ University of California at Santa Barbara, Santa Barbara, California 93106, United States; # QuidelOrtho, Inc., Rochester, New York 14626, United States; ∇ The Institute of Optics, University of Rochester, Rochester, New York 14627, United States; ○ ZDing Tech, LLC, Pittsford, New York 14534, United States

**Keywords:** ring resonator, coupling, fiber bundle, antibody assay, passive microfluidics, SARS-CoV-2

## Abstract

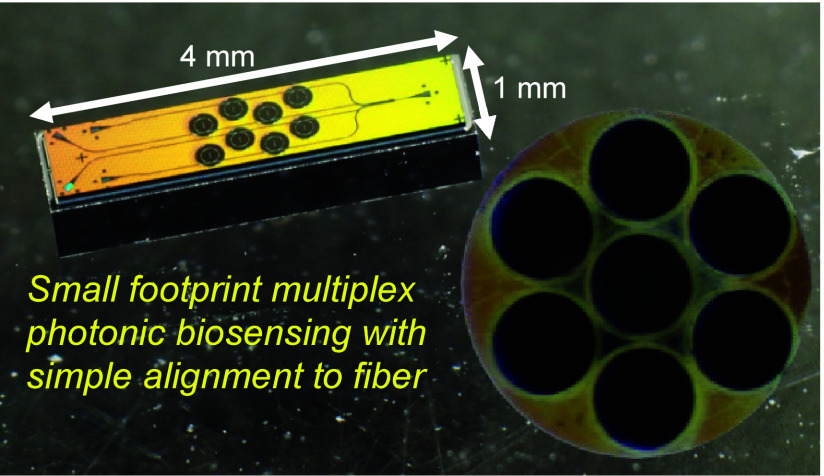

Photonic technologies promise to deliver quantitative, multiplex, and inexpensive medical diagnostic platforms by leveraging the highly scalable processes developed for the fabrication of semiconductor microchips. However, in practice, the affordability of these platforms is limited by complex and expensive sample handling and optical alignment. We previously reported the development of a disposable photonic assay that incorporates inexpensive plastic micropillar microfluidic cards for sample delivery. That system as developed was limited to singleplex assays due to its optical configuration. To enable multiplexing, we report a new approach addressing multiplex light I/O, in which the outputs of individual grating couplers on a photonic chip are mapped to fibers in a fiber bundle. As demonstrated in the context of detecting antibody responses to influenza and SARS-CoV-2 antigens in human serum and saliva, this enables multiplexing in an inexpensive, disposable, and compact format.

While research laboratories rapidly adopted methods such as the Luminex XMAP system for multiplex analysis of biological targets, the overwhelming majority of clinical analyzers remain limited to measuring a single analyte per sample.^1^ This is in part due to the challenge of obtaining FDA approval for multiplex diagnostics, but it is also a technological and cost challenge.^2^ Multiplex diagnostic systems often require complex fluid-handling subsystems,^3^ complex disposables,^4^ and complex instrumentation for readout. While progress has been made on simplified approaches to fluorescence-based multiplex assays,^5^ new approaches to multiplexing are a significant need.

We have previously described development of a “disposable photonics” sensor system, and its application for rapid detection of anti-SARS-CoV-2 antibodies in human serum.^6^ The disposable photonics platform integrates a grain of rice-sized (1 × 4 mm) ring resonator^7,8^ chip with a plastic micropillar microfluidic card enabling passive fluid transport (no power or pumps). Light input/output (I/O) is accomplished via vertically focusing grating couplers on the sensor chip. Both the assay consumable and light I/O system were designed with a focus on minimizing cost and maximizing the simplicity of operation. However, the system as designed was capable of reading only the output of a single photonic waveguide, severely limiting multiplex capability. While additional waveguides could be added to the 1 mm × 4 mm photonic chip without difficulty, it was not obvious how the outputs of each waveguide could be read without significantly increasing the complexity of the optical system and the experimental challenge of optical alignment.

In the literature, there are generally five methods of coupling light into and out of multiple waveguides and salient examples of each: (1) butt-coupling a fiber or fiber array to the waveguide edge facet,^9,10^ (2) free-space coupling from a lens to the waveguide edge facet,^11^ (3) coupling through a prism,^12,13^ (4) grating coupling from a fiber or fiber array,^14^ and (5) direct flip-chip bonding of a source.^15,16^ However, these approaches are either permanent (i.e., not compatible with high-volume production of an inexpensive disposable, at least given current technology), complex, expensive, or time-consuming to align. For clinical biosensing applications, it is desirable to facilitate facile, transient alignment between a reusable source/detector and a low-cost, low-complexity disposable sensor.

Here, we present a solution to that challenge in which each output grating from a waveguide is mapped through free-space optics to a single multimode fiber in a fiber bundle. The hexagonal close packing of output fibers into a bundle maximizes the count of outputs for a given ferrule diameter (1, 7, 19, etc.), while ensuring a consistent intercore distribution that can be mapped to gratings on the surface of a photonic integrated circuit (PIC). This significantly simplifies the optical alignment challenge for a multiplex sensor relative to the use of edge coupling or mapping light from grating couplers to a linear fiber array. We demonstrate the effectiveness of this approach via assays designed to measure human antibody responses to antigens from several SARS-CoV-2 variants as well as influenza antigens. While the COVID-19 pandemic is now officially “over”, immune status monitoring remains a useful tool for understanding vaccine efficacy as well as in the context of disease surveillance.^17^ This is particularly true as new SARS-CoV-2 variants emerge. These assays also serve as representative of a much broader set of clinically relevant assays.

## Methods

### Materials

Recombinantly expressed (baculovirus) SARS-CoV-2 antigens (RBD, N, S1 + S2), Influenza A and B antigens (HA), and monoclonal antibodies were obtained from Sino Biological, Inc. (Wayne, PA). Antifluorescein (anti-FITC) antibody used as a nonspecific binding control was obtained from Rockland Immunochemicals (Limerick, PA). The diluent for antibody/antigen printing was modified (potassium-free) phosphate-buffered saline (mPBS). Assay wash buffer (AWB), which was used to dilute serum samples, consisted of mPBS with 3 mM EDTA and 0.01% Tween-20. All serum samples were diluted 1:5 or higher, as noted, in AWB or AWB plus 4% fetal bovine serum (FBS; for samples diluted more than 1:10). (3-Glycidyloxypropyl)trimethoxysilane (GPTMS) was obtained from Gelest, Inc., Morrisville, PA. StabilGuard Immunoassay Stabilizer was obtained from Surmodics IVD Inc., Eden Prairie, MN.

Serum and saliva samples were obtained under a Dermatology Department Assay Development protocol approved by the University of Rochester Medical Center Institutional Review Board. All subjects were at least 18 years of age at the time of blood draw, subject to informed consent, and at least 14 days out of active disease. Whole blood samples were allowed to clot for 30 min after draw. Samples were then spun at 1200 × g for 5 min, and serum was pipetted off into a 15 mL conical tube and spun again for 10 min to remove any remaining cellular material. The serum was then aliquoted and stored at – 80 °C until use. Saliva samples were centrifuged for 2 min at 2400*g* then diluted 1:10 in AWB. These samples were not stored but were used immediately.

### Microring Resonators

Silicon nitride ring resonators were designed to interface with an upper aqueous cladding for use in biosensing. The ring resonators studied in this work consist of silicon nitride waveguides 1.5 μm wide and 220 nm tall, supporting a single transverse electric (TE) polarization mode. Modeling was performed by using the finite difference (FD) method in OptoDesigner, a component of the Synopsys Photonic Design Suite.

**Figure 1:**
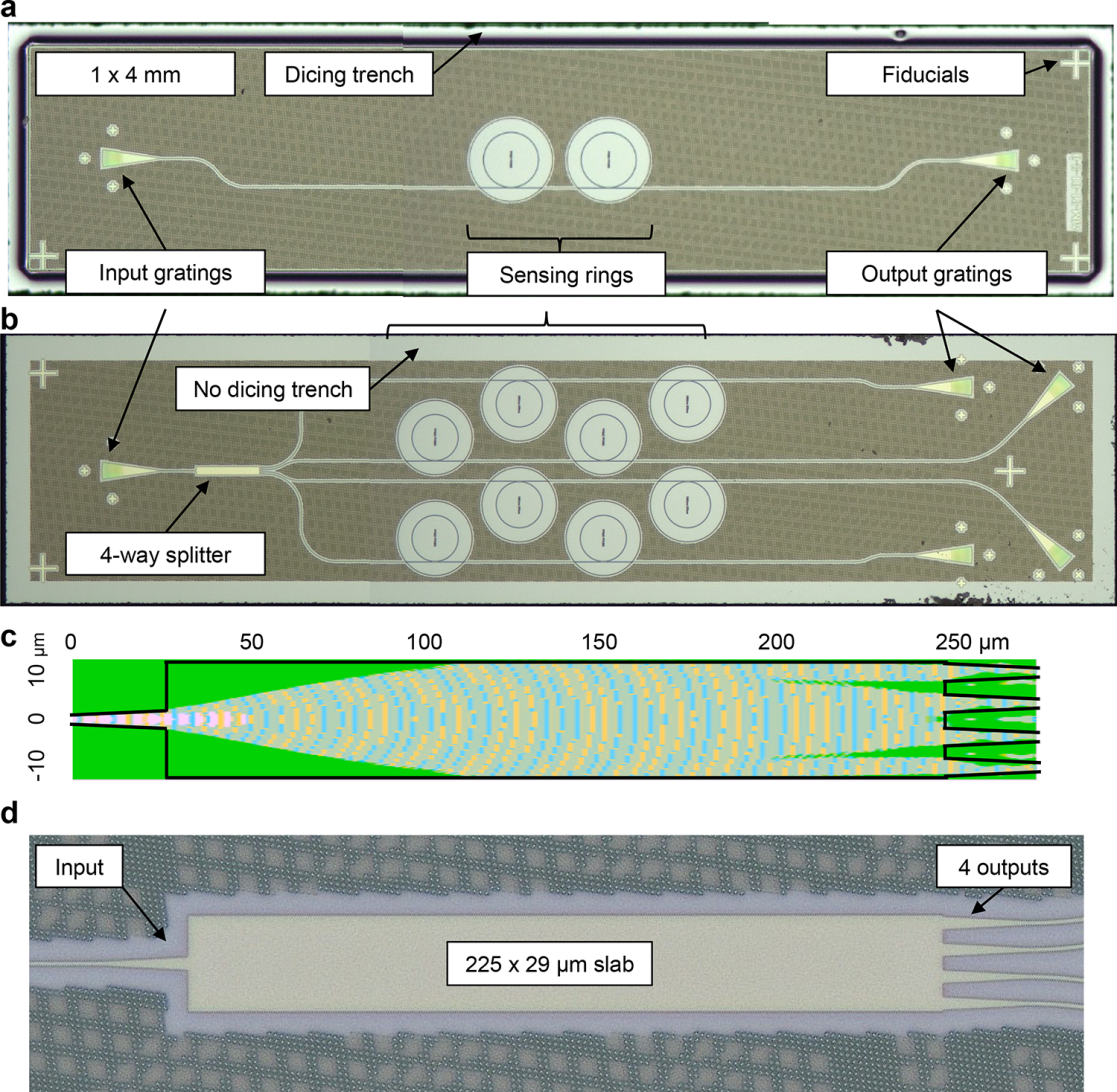
(a) Image of fabricated and diced singleplex PIC previously reported. Each PIC is 1 × 4 mm, including (in some examples) a 100 μm wide circumferential dicing trench. The 1 × 4 mm singleplex (1 experimental + 1 control) PIC design features a single input grating, 2 exposed rings evanescently coupled to a bus waveguide, and a single output grating. Fiducial marks on the PIC surface are provided for a potential future automated alignment system. (b) Image of the fabricated and diced multiplex PIC (this work). The 1 × 4 mm multiplex PIC design features a single input grating, a four-way multimode interference (MMI) splitter, eight microring resonators (2 per bus waveguide), and four output gratings. The dicing trench was omitted to improve fluid flow when packaged with micropillar microfluidics. (c) Finite-Difference Time-Domain (FDTD) modeling of 4-way splitter performed using Synopsys’ RSoft FullWave. Overall outline of the structure is shown in black; the input is at the left, and outputs are at the right. Calculated amplitude of interference field is shown in yellow/blue. (d) Micrograph of fabricated 4-way splitter.

Detailed descriptions of the layer stack and modeling of microring resonators have been reported previously, with the exception of an increase in the bottom oxide thickness from 5 to 5.3 μm to improve grating performance.^6^ For this study, each photonic integrated circuit (PIC) chip contains eight exposed rings for sensing. To fit eight rings within the 800 μm wide usable surface of the PIC, the ring diameter was decreased relative to our previous work from 198 to 164 μm and the coupling gap was decreased to 375 nm to compensate for increased bending losses and maintain near-critical coupling (Figure 1). Sensor PICs were designed to have two rings per bus waveguide, with each of the two rings having a slightly different diameter to yield resonance signals at different wavelengths based on the resonance condition λ = (), where *d* is the diameter of the ring.

### Multimode Interference Splitters

A four-way multimode interference (MMI) splitter was designed for 1550 nm wavelength light using Synopsys RSoft FullWave 2.5-D modeling (Figure 1c). The silicon nitride slab was 225 μm long and 29 μm wide. Input and outputs used a 25 μm taper from a 4 μm aperture to a 1.5 μm wide waveguide. A micrograph of the fabricated 4-way splitter is shown in Figure 1d.

### Grating Coupler Design

The grating coupler design has been described in detail previously.^6^ In brief, we incorporate a two-layer nitride approach, wherein the first nitride layer is the waveguide layer that transports the bulk of the electromagnetic energy. The second nitride layer comprised the periodic structures that form the grating. The nitride layers are separated vertically by a 100 nm oxide layer. The grating spacing is calculated to satisfy the following phase-matching condition to provide a spherical wave (eq 1):
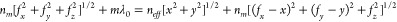
1where *f*_*x*_, *f*_*y*_, and *f*_*z*_ = 500 μm are the coordinates of the design focal point relative to the grating origin. The index of refraction of the medium is denoted by *n*_*m*_, while the effective index of the grating structure is denoted by *n*_*eff*_. The parameter *m* takes on integer values from 125 to 200.

The grating was designed by numerically simulating the Helmholtz equation in COMSOL Multiphysics V6.0. We used the finite element method in the *x-y* plane to determine suitable values for the parameter *m*. In this design, we chose not to use apodization. The results of the electromagnetic simulation for the *x-y* plane are shown in Figure S1. The origin of the grating is located at (*x,y*) = (0,0). A spherical wave is scattered by the grating to focus above the photonic chip surface at *f*_*z*_ = 500 μm. The beam waist at the focus region is diffraction limited to be approximately *d*_s_ = 15 μm and is approximately Gaussian.

Gaussian beam optics are positioned to be confocally aligned with the grating coupler at *f*_*z*_ = 500 μm. This provides standoff coupling between the external Gaussian optics and the photonic chip surface while minimizing inadvertent contact between the external optics and the PIC. On the output side of the PIC four gratings were positioned such that they would output light to the four uppermost and lowermost fibers of a custom 7-fiber bundle that will be described in more detail below.

### Photonic Chip Fabrication and Functionalization

Photonic sensors were fabricated using the 300 mm AIM Photonics fabrication line^18^ (Albany, NY) with modifications to the standard AIM passive multiproject wafer (MPW) process and layer stack described above. Images of the fabricated and diced chips are provided in Figure 1. Following fabrication, wafers were diced by the AIM Photonics Testing and Packaging Facility (Rochester, NY). Prior to functionalization, sensor PICs were removed from the dicing tape and first washed for 15 min in a 1:1 mixture of methanol and concentrated hydrochloric acid, then washed for 15 min in 3:1 mixture of concentrated sulfuric acid and 25% hydrogen peroxide (“piranha” solution; Caution! Piranha solution is highly caustic and reacts violently with organics), then rinsed 5 × 30 s in Nanopure water and dried with nitrogen. PICs were next placed in a chemical vapor deposition (CVD) oven (Yield Engineering Systems, Fremont, CA), where a monolayer of GPTMS was deposited on the surface.

Antigens and control antibodies were covalently attached to the functionalized surface by spotting them directly on the rings using a sciFLEXARRAYER SX piezoelectric microarrayer (Scienion AG, Berlin, Germany), using the manufacturer’s Find Target Reference Points (FTRP) machine vision protocol to accurately locate the position of the rings. The control rings were spotted with anti-FITC antibody at 550 μg mL^–1^ in mPBS (pH 7.2), and the test rings were spotted with SARS-CoV-2 receptor-binding domain (RBD) peptides, SARS-CoV-2 N-protein, or influenza A or B hemagglutinin at 400 μg mL^–1^ in mPBS (pH 7.2). The configuration for PICs functionalized with only RBD and anti-FITC is shown in Figure S2a. The configuration for PICs functionalized with all SARS-CoV-2 probes is presented in Figure S2b. All rings received approximately 3 nL of antibody/antigen solution. Chips were maintained at 75% humidity for 3 h, then overspotted with an equivalent volume of stabilizer solution (StabilGuard Immunoassay Stabilizer, Surmodics, Inc.). An image of the PICs after StabilGuard has been applied is shown in Figure S2c. Twenty minutes after stabilizer was spotted onto the rings, PICs were removed from the arrayer and kept in a desiccator until use.

### Assay Consumable Assembly

**Figure 2:**
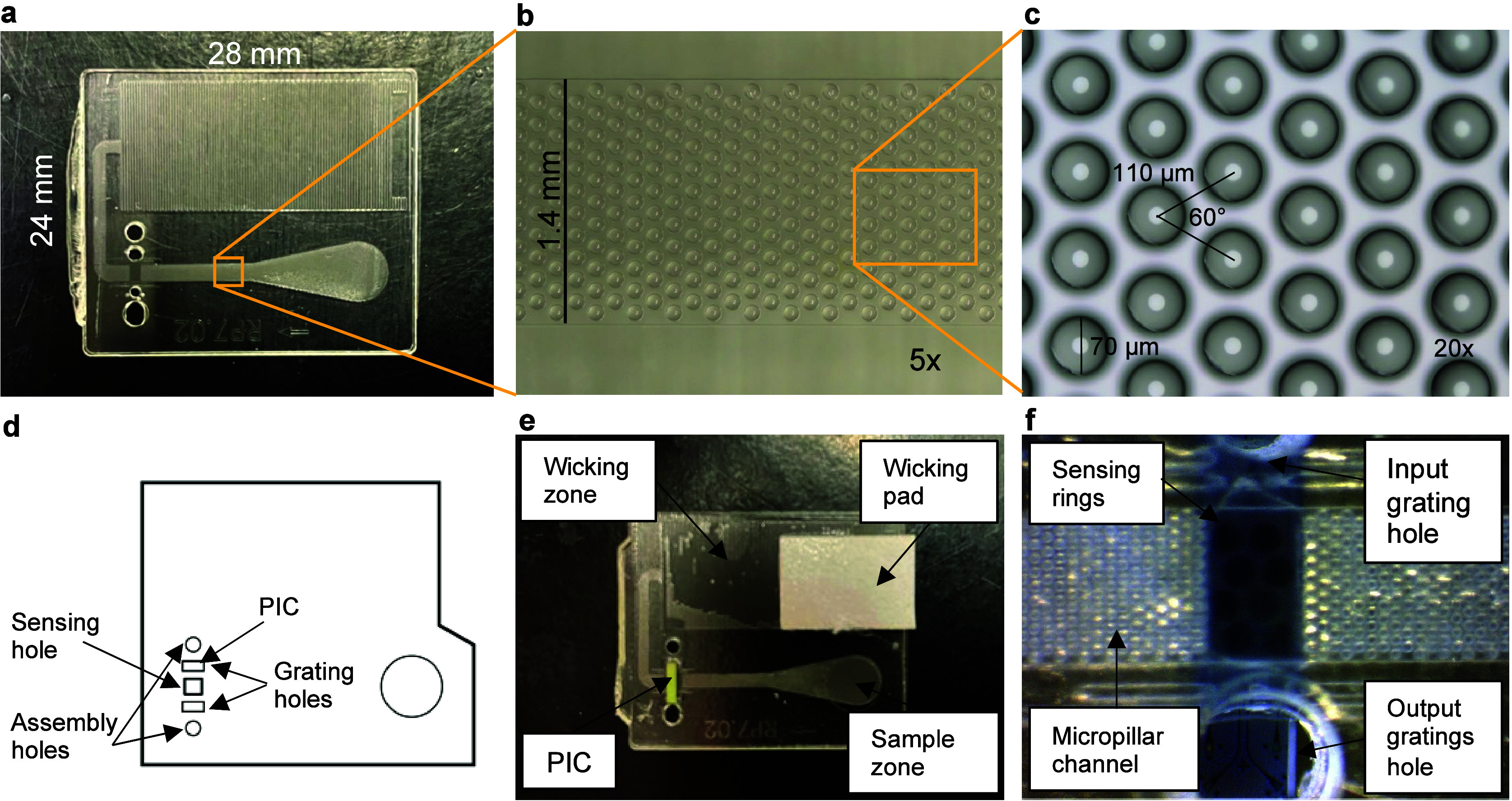
Multiplex PIC mounted to micropillar microfluidic card. (a) Image of a 24 mm × 28 mm micropillar card. (b) 5× enlargement of 1.4 mm wide micropillar channel between sample addition zone and PIC. (c) 20× enlargement of 70 μm diameter micropillars in hexagonal configuration with 110 μm pitch. (d) Layout of 3 M adhesive layer used to adhere PIC to the micropillar card that includes pass-throughs for gratings, microrings, assembly holes, and sample zone. (e) PIC mounted to a fully assembled micropillar card with adhesive tape and Whatman wicking pad. Sample is applied in the sample zone, which flows under the PIC and into the wicking zone reservoir. A Whatman wicking pad enables assays to be run with larger sample volumes than the current micropillar reservoir allows. (f) The multiplex PIC is aligned to the input and output optical pass-through as well as the microfluidic channel of the micropillar card.

PICs were integrated with an inexpensive microfluidic card designed to provide passive flow of sample liquids to the photonic chip for analysis. For precise control of analyte delivery, the microfluidic card requires a sample introduction zone, channels to direct fluid flow, a detection zone at a specified location over the channel where the PIC chip comes in contact with fluid, and a wicking zone to serve as a fluid sink and to drive fluid flow through a capillary action. In our previous work, prototype cards were fabricated by using a hot embossing process. For this work, the 24 × 28 mm polystyrene cards were produced by Syntec Optics (Rochester, NY) using an injection process. To fabricate the injection mold, the microfluidic card design was first patterned onto a silicon wafer by using photolithography and then etched by using reactive ion etching. The wafer was converted to a nickel-based electroform by electroplating (NiCoForm, Inc.). The nickel electroform was used to prepare the mold for the injection molding of fluidic cards. Each resulting card consists of a 1.4 mm width × 60 μm depth channel of pillars in a hexagonal configuration with 70 μm diameter and 110 μm pitch (Figure 2) except under the PIC, to avoid interruption of flow profile. The channel length from the sample zone to the wicking zone is about 20 mm. In the 10.75 mm × 22.5 mm wicking zone, the pillar diameter is 75 μm, and the height remains at 60 μm. Pillars are arranged in a diamond shape inside the wicking zone, with a pillar distance of 125 μm within each row and a 225 μm pitch across rows. In this pillar arrangement, fluid will advance in the wicking zone in a row-by-row fashion according to COMSOL simulation (Figure S3), reducing the risk of trapping bubbles. The total void volume of the wicking zone is 10.46 μL. In current design, the pillar matrix in the long channel contributes most of the flow resistance (>90%) in the fluidic pathway while the wicking zone offer an almost constant driving pressure for the fluid flow. The design is to achieve two different objectives: (1) to allow for at least five minutes flow time before wicking zone is filled by the sample with typical viscosity (e.g., serum at viscosity of 1.6 cps) given the current wicking zone size, pillar arrangement, and the surface properties (contact angle at 45°); (2) to generate an almost constant flow rate for in filling the wicking zone while minimizing the chance of trapping bubbles. Compared to our previously published work, the new cards have a larger diameter optical pass-through to accommodate additional gratings on the output side of the PIC. Polystyrene micropillar fluidic cards were treated immediately before assembly of the consumable with UV/Ozone for at least ten minutes to increase the hydrophilicity of the fluidic path (Novascan PSD Pro, Novascan Technologies, Inc., Boone, IA). The PIC was then bonded to the micropillar card by a patterned layer of double-sided, 57 μm-thick adhesive tape (467MP, 3M, St. Paul, MN). As shown in Figure 4d, holes in the adhesive tape for optical pass-through, assembly, sample addition, and sample interface with the PIC were patterned using a laser cutter (Full Spectrum Laser, Hobby Series 20 × 12, Las Vegas, NV). An image of the PIC positioned in the detection zone is shown in Figure 2e. An image of the underside of the assembled PIC and card reveals the microrings aligned to the channel and the gratings to their respective holes (Figure 2f).

Patterned adhesive tape was added to the fluidic cards using a custom alignment device, and a strip of filter paper (Q1, Whatman, Little Chalfont, UK) was placed between the micropillar outlet channel and adhesive to facilitate continuous flow once the channel had filled. Once the adhesive was applied to the fluidic card, photonic chips were manually aligned, aided by a custom jig to the channel and optical-access ports.

### Apparatus

The previously reported singleplex hub allowed for coupling to a single input and a single output grating. This limited the multiplex capability of the design as it is challenging to interpret spectra with multiple sensing rings per bus waveguide. Keeping the 1 mm × 4 mm PIC geometry and micropillar channel constant, we found that the number of rings could be readily expanded to eight (and potentially more) by incorporating four bus waveguides onto the PIC. By using a four-way splitter, the input of the optical hub and PIC could remain unchanged. However, this required a different design for the output to capture light from four or more distinct gratings. We addressed this need by using a fiber bundle as that would yield a reproducible configuration of close-packed output fibers. Combined with positioning the output gratings to match the configuration of the fiber bundle and a new lens design to image the gratings on to the bundle with the appropriate magnification, we hypothesized that such an approach would bring the advantages of the disposable photonics assay system to multiplex analyses.

**Figure 3:**
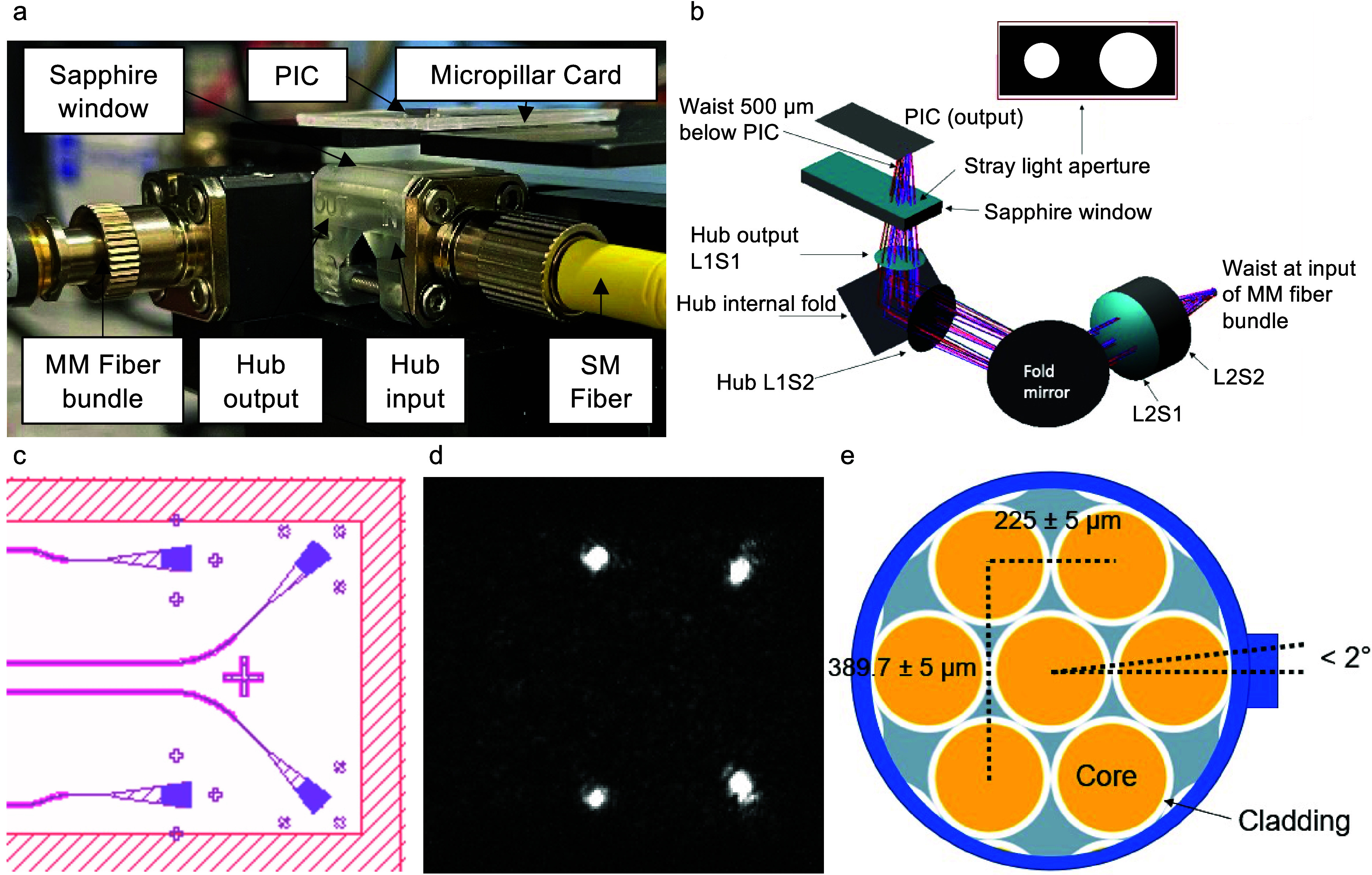
(a) Side view of a PIC/micropillar card aligned to the multiplex optical hub. Input light arrives via a single-mode (SM) fiber on the right and passes through the input optics of the hub to the PIC. (b) Ray diagram of the output optical lens system. Light emitted from the output gratings of the PIC passes through the first surface of the hub output lens L1S1, reflects 90° off the angled interior surface of the hub, and exits the second surface L1S2 into free space as a collimated beam. A fold mirror is employed to reflect the beam 90° toward lens L2, which focuses the beam on to facets of a custom 7-fiber multimode fiber bundle. (c) Output gratings of the multiplex PIC correspond to the illuminated gratings in the IR micrograph in (b). (d) IR micrograph of the four output gratings of a PIC with the light coupled through the input. (e) End-on diagram of custom 7-fiber bundle with hexagonal close-packing. Two uppermost and lowermost multimode fibers collect light from the four output gratings of the PIC.

The execution of this concept is shown in Figure 3. The assembled assay consumable was aligned to an optical source, which consists of a custom multiplex optical element (Syntec Optics, Rochester, NY) that enabled light to be coupled to and from the photonic grating couplers from below the micropillar card. A side view of the multiplex optical hub apparatus is shown in Figure 3a. While the input optical design is identical to the previously reported singleplex hub, the output optical design was modified to enable an interface with four or more gratings instead of one. A ray diagram of the modified output optical design is shown in Figure 3b (optical simulation by Moondog Optics, Fairport, NY). A folded mirror was incorporated into the output side to keep the overall scale of the system compact. The modified output lens system is designed to image the four output gratings of the multiplex PIC (Figure 3c; output light visualized via an IR microscope in Figure 3d) to the upper two and lower two fibers of a custom multimode fiber bundle (Idil Fibres Optiques, France) shown in Figure 3e. Additional gratings can of course be used to enable up to 7 outputs, given the use of the current 7-fiber bundle.

A schematic of the complete measurement apparatus is presented in Figure S4. A tunable laser source (Keysight 81606A) is directed through a polarization controller (Thorlabs FPC561 with SMF-28 FC/PC connectors) to obtain linearly polarized light with TE orientation relative to the silicon nitride waveguide. Light is directed through the input of the optical hub (15 × 15 × 16 mm; currently diamond-turned by Syntec Optics, Rochester, NY, although injection molding may be possible) and focused on the input grating of the PIC. Output light from the four PIC output gratings is collected by the optical hub and directed through a custom fiber bundle (IDIL Optics) of multimode fibers (Thorlabs FP200ERT) to four channels of the optical power meter (Keysight N7745A). Alignment of the PIC to the optical hub is facilitated by a dual-camera VIS/IR microscope. A 5× IR objective lens (Mitutoyo Plan Apo NIR 46–402) with on-axis illumination directs light though a long-pass dichroic mirror (Thorlabs DMLP950R) to either the IR camera (WiDy InGaAs 650) or VIS CMOS camera (Thorlabs DCC1645C). Proper alignment is confirmed by IR micrograph and resonance spectra.

The tunable laser and optical power meter are connected to a computer via a general-purpose interface bus (GPIB) and are controlled by the Insertion Loss software of the Keysight Photonic Application Suite (N7700A). Spectral measurements were recorded by repeated wavelength sweeps. Ten nm spectra were taken continuously at 1 pm resolution, centered on 1550 nm, with each spectral sweep taking about 6 s. All spectra were automatically saved for analysis. Once a spectrum was acquired after alignment, samples were added at appropriate dilutions were added directly, without a prewash. The resonance red-shift is proportional to the binding of material to the ring surface. Specific shift due to capture of target analyte is calculated by subtracting the redshift of the control ring from that of the probe ring, using a data analysis protocol discussed below.

### Analysis of Spectra

Collected spectra were processed automatically through custom software written in Python and deployed on Anaconda. The output spectra for each channel were collected simultaneously and stored in the same file. Minor modifications were made to the previously described pipeline to accommodate multiple output channels. Briefly, spectral features including peak location, peak height, quality factor, chi-squared values, and peak fitting parameters are extracted by fitting the data with a Lorentzian function. These data for each peak in the spectrum as a function of time are output in a CSV format for subsequent analysis.

## Results

### Performance of Multiplex Hub

Performance of the multiplex hub was found to be comparable to that of the previous singleplex hub. When output gratings of a multiplex PIC (Figure S5a) were properly aligned with the corresponding output fibers of the bundle (Figure S5b), insertion loss of the entire system was ∼20 dB. Axial rotation of the fibers with the bundle ferrule was, as expected, a critical parameter to efficient capture of output light from each grating. Coupled power was found to decrease precipitously beyond ∼5° axial rotation. An end-on view of a properly configured fiber bundle is shown inFigure S5c.

A major goal during the design of the multiplex hub was to preserve backward compatibility with earlier PIC designs for the singleplex hub. This is accomplished by capturing the output light from a singleplex PIC with the central fiber of the fiber bundle. Using this approach, we found the singleplex PIC output power to be equivalent to our previous system.

### Characterization of Fabricated Photonic Sensors

**Figure 4:**
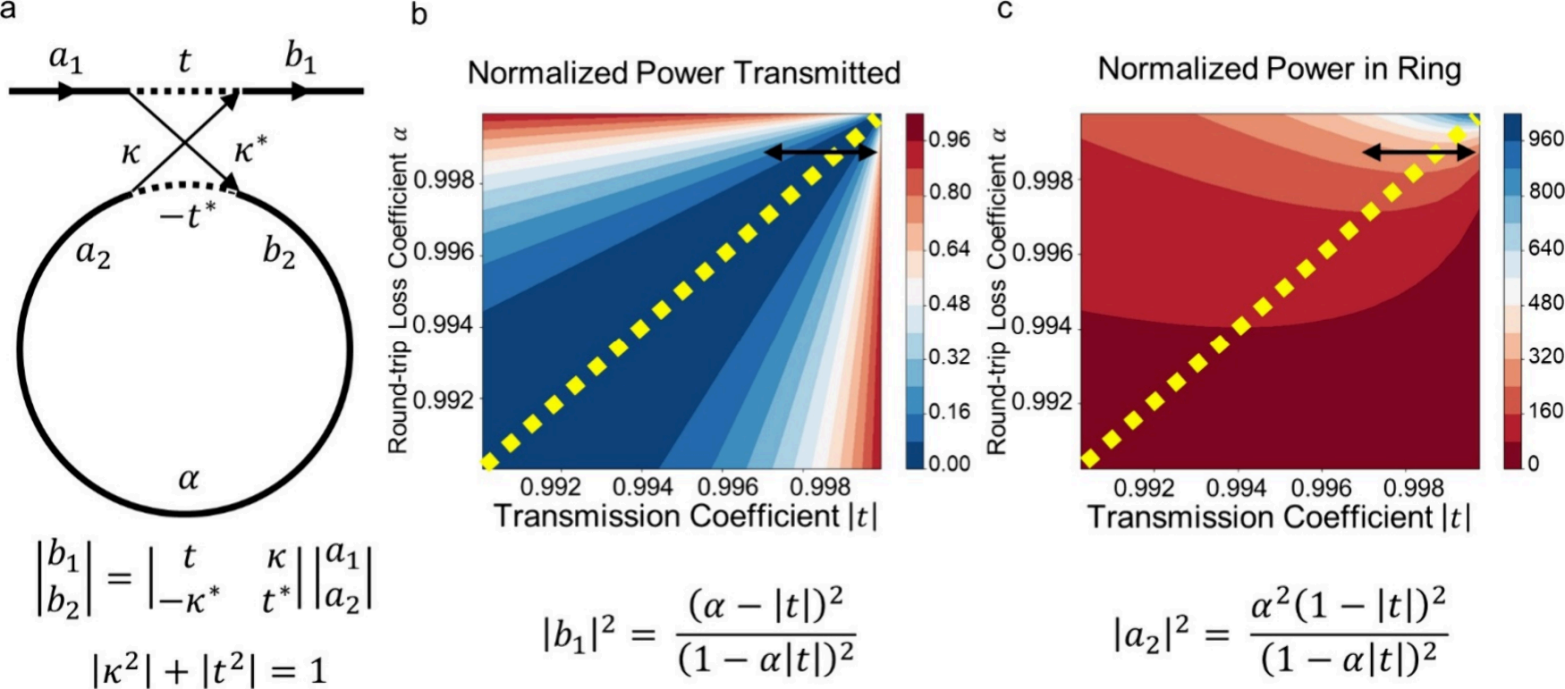
(a) Ring resonator relations as described by Yariv^19^ The normalized interaction between bus waveguide and ring resonator is described by the matrix relation mapping the complex amplitudes of guided modes in the bus and ring waveguides a_1_ and a_2_, to the transmitted and coupled amplitudes b_1_ and b_2_. The coupling between waveguide and resonator is dictated by transmission and coupling coefficients t and κ and made lossless by requiring that the sum of their square magnitudes is identical to one. The internal loss of the ring is defined by the round-trip loss coefficient α, which describes a lossless ring when it is identical to one. (b) Normalized power transmitted to the output ring resonator-coupled bus waveguide is inversely proportional to the extinction ratio (ER), shown here as a function of transmission and round-trip loss coefficients. Critical coupling that results in zero transmitted power and the maximum ER is indicated by the dashed yellow line where transmission and round-trip loss coefficients are identical. The black double arrow illustrates the latitude of normalized transmitted power resulting from fabrication variations that affect the coupling and transmission coefficients. (c) Normalized power in the ring is proportional to the quality factor. The resulting Q-factor at critical coupling is indicated by the yellow dashed line. The double black arrow indicates the range of expected Q-factors resulting from fabrication variations.

A design constraint of the present system was that the diameter of the multiplex rings had to be reduced relative to our previous work to fit 8 rings within the footprint of the same 1 × 4 mm PIC. We observed that resonance peaks for fabricated 8-ring PICs are easily resolved from signal background due to high quality factors and significant extinction ratio. The measured quality factors (Q) and extinction ratios typically exceeded 4 × 10^4^ and 20 dB, respectively. Previously, we have reported state-of-the-art quality factors for silicon nitride ring resonators with an aqueous cladding used for biosensing.^6^ However, achieving the greatest quality factor attainable for a resonator, above a certain threshold, may not be the best approach for applied biological sensing. When analyzing the effect of ring resonator coupling, wavelength, and loss on the quality factor and extinction ratio (Figure 4a), it is apparent that greater quality factors can be achieved by decreasing the loss in the ring (Figure 4c). Yet, as ring loss decreases, the effect on the extinction ratio of changing wavelength and fabrication variations to the coupling gap becomes more severe (Figure 4b). Therefore, introducing more ring loss by decreasing the ring diameter yields a ring resonator that is more tolerant to fabrication variation, with the ancillary benefit that it also has a smaller footprint. Another benefit to a smaller ring diameter is the corresponding increase in the free spectral range (FSR) between sequential resonance peaks, as well as a more favorable extinction ratio over a broad range of wavelengths (Figure S6). The FSR for the multiplex-configuration rings was 2.54 at 1555 nm vacuum wavelength, compared to 2.14 nm for the singleplex-configuration rings. With extinction ratios exceeding 20 dB under aqueous cladding, these rings are well designed to enable sensing. No cross-talk between channels was observed for multiplex PICs (Figure S7).

### Assay Data

Multiplex capability enables the acquisition of assay results from replicate sensors on the same photonic chip (increasing statistical significance), detection of multiple analytes (increasing biological information density per ship), or a mixture of both. As a first test of the multiplex assay system, we printed one ring on each waveguide with wild-type Receptor Binding Domain (RBD) protein from SARS-CoV-2, and the other with an antibody to fluorescein (anti-FITC) as a negative control (print layout as shown in Figure S2a). Eight replicate assays of a 5:1 dilution of serum from an individual known to have strong anti-RBD antibody response were run to assess intra- and interassay reproducibility. Typical spectra from one of these assays are presented in Figure S8. Each ring has a corresponding resonant wavelength at which we see a trough in transmitted power. The peaks on the left correspond to the anti-FITC-functionalized rings, and the peaks on the right correspond to the RBD peptide-functionalized rings. With the addition of the diluted human serum sample, the right peak shifts as antibodies bind to the ring while the anti-FITC ring shifts much less. The anti-FITC shift is nonzero due to nonspecific interactions with serum proteins. From these experiments, an average anti-FITC corrected shift of 496.8 ± 41.6 pm (standard deviation) was observed for the RBD-functionalized rings across all replicates. Overall inter- and inter-run CVs are low (Table 1) with the exception of one replicate. This suggests that the approach is able to provide reproducible assay results, at least for a high-concentration analyte such as anti-RBD in a vaccinated or recently infected individual. High intrarun CV in run 5 may have occurred due to introduction of debris on the sensor in the consumable assembly process.

**Table 1:** Results from 8 Replicate Runs of a Single-Donor Human Serum Sample^a^

**run**	**net RBD shift**	**R1**	**R2**	**R3**	**R4**	**intrarun CV (%)**
1	504 ± 13	535	550	551	515	3.1
2	516 ± 9	530	560	559	519	3.8
3	514 ± 13	565	548	545	525	3.0
4	504 ± 18	547	566	544	545	1.9
5	478 ± 27	362	640	529	528	22.7
6	513 ± 8	555	567	532	501	5.4
7	542 ± 12	581	598	584	525	5.6
8	421 ± 15	485	511	467	445	5.9
inter-run CV (%)		13.6	6.7	6.3	5.9	

a All resonance shifts are reported in pm. “Net RBD shift” indicates the value obtained after subtraction of the anti-FITC response for that run, with error propagated for both RBD and anti-FITC measurements. R1, R2, R3, and R4 represent shifts for individual RBD rings for that run.

Next, two pools of normal human serum were prepared, one from a group of 15 subjects all one week post a second dose of the Pfizer mRNA SARS-CoV-2 vaccine with no history of COVID-19 disease, and another from a group of 10 subjects all postpositive PCR test with varying vaccination status. Serial dilutions of the pooled samples were prepared (1:10, 1:100, 1:1000, and 1:10,000, 1:100,000, and 1:1,000:000), and run on an 8-plex assay (print layout as shown in Figure S2b) consisting of SARS-CoV-2 WT RBD, several RBD variants, WT Nucleocapsid (N) protein, and once again anti-FITC as control. To ensure the highest quality response for highly dilute samples, the assay time was increased to 10 min. Each sample was run in a quintuplet. All analytes were analytically well behaved on the 8-plex assay (Figure S9a,b), demonstrating dose–response curves titrating to zero with dilution. As expected, vaccinated subjects’ pooled serum displayed strong antibody binding to WT RBD as well as RBD variants, with no anti-N protein response, while the pooled serum derived from subjects post-COVID also showed an anti-N response. Limits of detection (LOD) for the 7 analytes were calculated^20^ and are displayed in Table S2. While these LOD values are strongly influenced by the immune responses of individuals making up the pooled sera and are thus not informative in terms of sensor performance, they nevertheless reveal interesting differences between vaccinated groups and those recently subject to SARS-CoV-2 infection. Importantly, LLODs measured for the pooled vaccinated serum anti-WT RBD responses were within experimental error in the multiplex format (1:106,402 dilution) and singleplex format (1:111,760 dilution), indicating the increase in multiplex capability did not carry any performance penalty. These detection limits are comparable to if not better than those reported for other multiplex techniques including plasmon-enhanced fluorescence.^21^ Experiments with monoclonal antibodies against WT N showed no cross-reactivity with WT or delta RBD, while a monoclonal antibody against WT RBD cross-reacted with delta RBD but only to a limited extent with other mutant RBD proteins (Figure S11).

**Figure 5:**
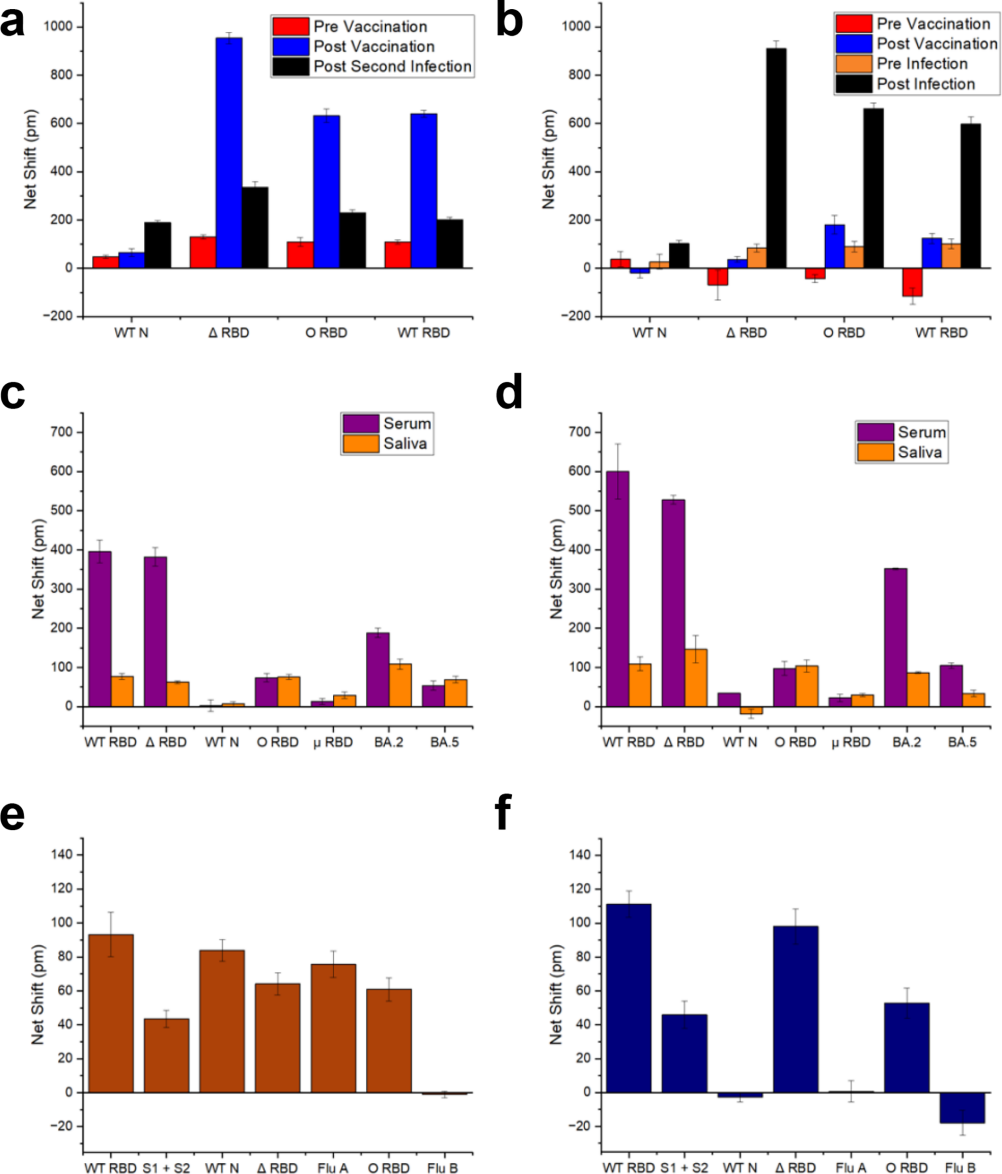
Representative multiplex assay results on single-donor samples. (a, b) Average anti-FITC corrected shifts for 4-plex assays run on longitudinal samples from two individuals. Results represent 8 replicate runs of each sample, with standard deviations shown. (c, d) Comparisons of responses obtained on 7-plex assays for two individuals, comparing serum (purple) and saliva (orange) samples. Results represent 8 replicate runs of each sample, with standard deviations shown. (e, f) Multiplex analysis can also report on immune status for other upper respiratory viruses. The subject in (e) was PCR-positive for SARS-CoV-2 and had a history of influenza A exposure, while the subject in (f) was PCR-positive, recently vaccinated with the Pfizer mRNA vaccine, and had no influenza exposure history.

Next, we examined multiplex responses for individuals. Longitudinal samples for two donors (Figure 5a,b) who had been vaccinated against SARS-CoV-2 but also exhibited breakout infections with the virus were tested. Clear differences in antibody response are observed following vaccination, but also following breakout infections. As expected, and as in the pooled serum results discussed above, anti-N signals are only observed following episodes of COVID-19 disease. However, the anti-N response is muted in comparison to anti-RBD concentrations in post infection individuals. Cross-reactive responses to RBD for different variants after vaccination or infection are useful for understanding the level of protection for each variant, as previous work has shown a correlation between antibody affinity and neutralization potency.^22^

Several groups have also reported detection of antibodies in human saliva.^23,24^ To test the suitability of saliva on this platform, we examined paired saliva and serum samples from two individuals, neither of whom had a known history of COVID-19 disease. We found that the platform was readily able to detect antibodies in saliva (Figure 5 c,d). While differences in total antibody titer between serum and saliva were expected, differences in the relative amounts of antibodies binding variant RBDs between serum and saliva samples was unexpected. Other authors have observed this as well, and note that differences may be due to individual Ig isotype distributions.^25^

Finally, we tested the suitability of the platform for simultaneous detection of anti-SARS-CoV-2 and anti-influenza antibody response. PICs were derivatized with influenza A and B hemagglutinins (HA), and WT SARS-CoV-2 antigens RBD, N, and S1+S2, and tested with two single-donor human serum samples. One subject was PCR-positive for SARS-CoV-2, had no history of SARS-CoV-2 vaccination, and was known to have a history of influenza A infection, while the other was PCR-negative for SARS-CoV-2, had undergone a two-dose course of SARS-CoV-2 mRNA vaccination, and had no known history of either influenza A vaccination or infection. As shown in Figure 5e,f, the immunological histories of these two subjects with regard to SARS-CoV-2 and influenza A were accurately reflected in the multiplex sensor response.

## Conclusions

We have demonstrated expansion of an inexpensive disposable silicon photonics sensor platform to a multiplex solution integrating silicon nitride ring resonator-based biodetection with plastic micropillar microfluidics for sample handling. The key innovation making this possible is mapping output focusing gratings from the sensor PIC to the fiber bundle. Because the dimensions and geometry of the bundle are precisely defined by the size and packing geometry of the individual fibers, this presents a simple and highly reproducible solution to the problem of optical coupling. Alignment of sensors is dramatically faster in our hands using this approach than is possible with a fiber array (seconds vs minutes), which should simplify automation as well as point-of-care (PoC) solutions. This approach also provides further benefits: first, arranging gratings in 2D more efficiently uses chip real estate than a linear array. Second, as fibers self-assemble within a ferrule, fabrication of the coupling system is anticipated to be simpler and less expensive than that of an equivalent v-groove fiber array. Ring resonator PICs produced at 300 mm wafer scale using foundry CMOS processes (AIM Photonics) are of high optical quality and provide state-of-the-art biosensing performance when combined with a micropillar microfluidics system. Sensors functionalized with SARS-CoV-2 antigens (WT RBD, RBD variants, and nucleocapsid protein) can detect and quantify antibody responses in human serum and saliva samples. Importantly, sensor responses reflect known variation in the biological state for multiplex analytes.

Since medical diagnostics are typically single-use, cost considerations are of considerable importance in evaluating a new sensing system. Here, use of the plastic, passive microfluidic card, and small PIC help to keep the overall cost of the assay low, with an overall calculated Cost of Goods Sold (COGS) of $2.05 (Table S3). While COGS is only part of the final cost of an assay,^26^ it is nonetheless an important starting point for cost reduction. Use of passive microfluidics also helps to limit instrument complexity as it removes the need for powered fluid handling systems. We anticipate that the COGS for a PoC system can be in the $5,000 range and are currently working toward that goal.

Multiplex serological assays can play an important role, as a pandemic becomes an endemic. As novel variants continue to erupt into the population, it will be important to understand an individual’s cross-immunity profile to new variants. Such studies could aid in the choice of variants to be included in future booster vaccines. We anticipate that this platform will be suitable for use in many other diagnostic contexts, and efforts along those lines are in progress in our laboratories.

## Supporting Information Available

COMSOL model of microfluidic flow, apparatus schematic, images of fiber bundle and alignment, and results for pooled serum samples (PDF)

## References

[ref1] Vashist S. K.; Luong J. H. T. Chapter 1 - Immunoassays: An Overview. In Handbook of Immunoassay Technologies; Vashist S. K., Luong J. H. T., Eds.; Academic Press, 2018; pp 1–18 10.1016/B978-0-12-811762-0.00001-3.

[ref2] Tighe P. J.; Ryder R. R.; Todd I.; Fairclough L. C. ELISA in the Multiplex Era: Potentials and Pitfalls. Proteomics: Clin. Appl. 2015, 9 (3–4), 406–422. 10.1002/prca.201400130. 25644123 PMC6680274

[ref3] Donato L. J.; Theel E. S.; Baumann N. A.; Bridgeman A. R.; Blommel J. H.; Wu Y.; Karon B. S. Evaluation of the Genalyte Maverick SARS-CoV-2 Multi-Antigen Serology Panel. Journal of Clinical Virology Plus 2021, 1 (3), 100030 10.1016/j.jcvp.2021.100030. 35262016 PMC8213521

[ref4] Fanshawe T. R.; Glogowska M.; Edwards G.; Turner P. J.; Smith I.; Steele R.; Croxson C.; Bowen J. S. T.; Hayward G. N. Pre-Analytical Error for Three Point of Care Venous Blood Testing Platforms in Acute Ambulatory Settings: A Mixed Methods Service Evaluation. PLOS ONE 2020, 15 (2), e0228687 10.1371/journal.pone.0228687. 32012203 PMC6996845

[ref5] Qiu X.; Xu J.; Cardoso Dos Santos M.; Hildebrandt N. Multiplexed Biosensing and Bioimaging Using Lanthanide-Based Time-Gated Förster Resonance Energy Transfer. Acc. Chem. Res. 2022, 55 (4), 551–564. 10.1021/acs.accounts.1c00691. 35084817

[ref6] Cognetti J. S.; Steiner D. J.; Abedin M.; Bryan M. R.; Shanahan C.; Tokranova N.; Young E.; Klose A. M.; Zavriyev A.; Judy N.; Piorek B.; Meinhart C.; Jakubowicz R.; Warren H.; Cady N. C.; Miller B. L. Disposable Photonics for Cost-Effective Clinical Bioassays: Application to COVID-19 Antibody Testing. Lab on a Chip 2021, 21 (15), 2913–2921. 10.1039/D1LC00369K. 34160511

[ref7] Steglich P.; Hülsemann M.; Dietzel B.; Mai A. Optical Biosensors Based on Silicon-On-Insulator Ring Resonators: A Review. Molecules 2019, 24 ( (3), ). 519 10.3390/molecules24030519. 30709027 PMC6384601

[ref8] Meserve K.; Chapman C.; Escalante P.; Bailey R. C. B-036 Multi-Biomarker Approach to Latent Tuberculosis Diagnosis Using Microring Resonator Sensors. Clin. Chem. 2023, 69 (Supplement_1), hvad097.379 10.1093/clinchem/hvad097.379.

[ref9] Bryan M. R.; Butt J. N.; Bucukovski J.; Miller B. L. Biosensing with Silicon Nitride Microring Resonators Integrated with an On-Chip Filter Bank Spectrometer. ACS Sens. 2023, 8 (2), 739–747. 10.1021/acssensors.2c02276. 36787432 PMC9972465

[ref10] Cognetti J. S.; Moen M. T.; Brewer M. G.; Bryan M. R.; Tice J. D.; McGrath J. L.; Miller B. L. A Photonic Biosensor-Integrated Tissue Chip Platform for Real-Time Sensing of Lung Epithelial Inflammatory Markers. Lab on a Chip 2023, 23 (2), 239–250. 10.1039/D2LC00864E. 36594179 PMC10311125

[ref11] Tyndall N. F.; Stievater T. H.; Kozak D. A.; Koo K.; McGill R. A.; Pruessner M. W.; Rabinovich W. S.; Holmstrom S. A. Waveguide-Enhanced Raman Spectroscopy of Trace Chemical Warfare Agent Simulants. Opt. Lett., OL 2018, 43 (19), 4803–4806. 10.1364/OL.43.004803. 30272744

[ref12] Park D. S.-W.; Young B. M.; You B.-H.; Singh V.; Soper S. A.; Murphy M. C. An Integrated, Optofluidic System With Aligned Optical Waveguides, Microlenses, and Coupling Prisms for Fluorescence Sensing. Journal of Microelectromechanical Systems 2020, 29 (4), 600–609. 10.1109/JMEMS.2020.3004374. PMC11465942 39391841

[ref13] Safronov K. R.; Bessonov V. O.; Akhremenkov D. V.; Sirotin M. A.; Romodina M. N.; Lyubin E. V.; Soboleva I. V.; Fedyanin A. A. Miniature Otto Prism Coupler for Integrated Photonics. Laser & Photonics Reviews 2022, 16 (4), 2100542 10.1002/lpor.202100542.

[ref14] Duval D.; Osmond J.; Dante S.; Domínguez C.; Lechuga L. M. Grating Couplers Integrated on Mach-Zehnder Interferometric Biosensors Operating in the Visible Range. IEEE Photonics Journal 2013, 5 (2), 3700108–3700108. 10.1109/JPHOT.2013.2251873.

[ref15] Shi T.; Wang H.; Meng R.; Xu L.; Wang T.; Zheng W. Flip-Chip Bonded Evanescently Coupled III-V-on-Si Single-Mode Laser with Slotted Feedback Structure. IEEE Photon. Technol. Lett. 2021, 33 (14), 739–742. 10.1109/LPT.2021.3090565.

[ref16] Theurer M.; Moehrle M.; Sigmund A.; Velthaus K.-O.; Oldenbeuving R. M.; Wevers L.; Postma F. M.; Mateman R.; Schreuder F.; Geskus D.; Worhoff K.; Dekker R.; Heideman R. G.; Schell M. Flip-Chip Integration of InP to SiN Photonic Integrated Circuits. J. Lightwave Technol. 2020, 38 (9), 2630–2636. 10.1109/JLT.2020.2972065.

[ref17] Doppler C.; Feischl M.; Ganhör C.; Puh S.; Müller M.; Kotnik M.; Mimler T.; Sonnleitner M.; Bernhard D.; Wechselberger C. Low-Entry-Barrier Point-of-Care Testing of Anti-SARS-CoV-2 IgG in the Population of Upper Austria from December 2020 until April 2021—a Feasible Surveillance Strategy for Post-Pandemic Monitoring?. Analytical and Bioanalytical Chemistry 2022, 414 (10), 3291–3299. 10.1007/s00216-022-03966-z. 35229172 PMC8885117

[ref18] Fahrenkopf N. M.; McDonough C.; Leake G. L.; Su Z.; Timurdogan E.; Coolbaugh D. D. The AIM Photonics MPW: A Highly Accessible Cutting Edge Technology for Rapid Prototyping of Photonic Integrated Circuits. IEEE Journal of Selected Topics in Quantum Electronics 2019, 25 (5), 1–6. 10.1109/JSTQE.2019.2935698.

[ref19] Yariv A. Universal Relations for Coupling of Optical Power between Microresonators and Dielectric Waveguides. Electronics letters 2000, 36 (4), 321–322. 10.1049/el:20000340.

[ref20] Klose A. M.; Daiss J. L.; Ho L.; Beck C. A.; Striemer C. C.; Muthukrishnan G.; Miller B. L. StaphAIR: A Label-Free Antigen Microarray Approach to Detecting Anti-Staphylococcus Aureus Antibody Responses in Orthopedic Infections. Anal. Chem. 2021, 93 (40), 13580–13588. 10.1021/acs.analchem.1c02658. 34596381

[ref21] Cady N. C.; Tokranova N.; Minor A.; Nikvand N.; Strle K.; Lee W. T.; Page W.; Guignon E.; Pilar A.; Gibson G. N. Multiplexed Detection and Quantification of Human Antibody Response to COVID-19 Infection Using a Plasmon Enhanced Biosensor Platform. Biosensors and Bioelectronics 2021, 171, 112679 10.1016/j.bios.2020.112679. 33069957 PMC7545244

[ref22] Muecksch F.; Weisblum Y.; Barnes C. O.; Schmidt F.; Schaefer-Babajew D.; Wang Z.; C. Lorenzi J. C.; Flyak A. I.; DeLaitsch A. T.; Huey-Tubman K. E.; Hou S.; Schiffer C. A.; Gaebler C.; Da Silva J.; Poston D.; Finkin S.; Cho A.; Cipolla M.; Oliveira T. Y.; Millard K. G.; Ramos V.; Gazumyan A.; Rutkowska M.; Caskey M.; Nussenzweig M. C.; Bjorkman P. J.; Hatziioannou T.; Bieniasz P. D. Affinity Maturation of SARS-CoV-2 Neutralizing Antibodies Confers Potency, Breadth, and Resilience to Viral Escape Mutations. Immunity 2021, 54 (8), 1853–1868. 10.1016/j.immuni.2021.07.008. 34331873 PMC8323339

[ref23] MacMullan M. A.; Ibrayeva A.; Trettner K.; Deming L.; Das S.; Tran F.; Moreno J. R.; Casian J. G.; Chellamuthu P.; Kraft J.; Kozak K.; Turner F. E.; Slepnev V. I.; Le Page L. M. ELISA Detection of SARS-CoV-2 Antibodies in Saliva. Sci Rep 2020, 10 (1), 20818 10.1038/s41598-020-77555-4. 33257702 PMC7705674

[ref24] Casian J. G.; Angel A. N.; Lopez R.; Bagos C.; MacMullan M. A.; Bui M. L.; Chellamathu P.; Das S.; Turner F.; Slepnev V.; Ibrayeva A. Saliva-Based ELISAs for Effective SARS-CoV-2 Antibody Monitoring in Vaccinated Individuals. Front. Immunol. 2021, 12, 701411 10.3389/fimmu.2021.701411. 34539632 PMC8446671

[ref25] Dobaño C.; Alonso S.; Vidal M.; Jiménez A.; Rubio R.; Santano R.; Barrios D.; Pons Tomas G.; Melé Casas M.; Hernández García M.; Girona-Alarcón M.; Puyol L.; Baro B.; Millat-Martínez P.; Ajanovic S.; Balanza N.; Arias S.; Rodrigo Melero N.; Carolis C.; García-Miquel A.; Bonet-Carné E.; Claverol J.; Cubells M.; Fortuny C.; Fumadó V.; Codina A.; Bassat Q.; Muñoz-Almagro C.; Fernández De Sevilla M.; Gratacós E.; Izquierdo L.; García-García J. J.; Aguilar R.; Jordan I.; Moncunill G. Multiplex Antibody Analysis of IgM, IgA and IgG to SARS-CoV-2 in Saliva and Serum From Infected Children and Their Close Contacts. Front. Immunol. 2022, 13, 751705 10.3389/fimmu.2022.751705. 35154094 PMC8828491

[ref26] Wilson D. J.; Kumar A. A.; Mace C. R. Overreliance on Cost Reduction as a Featured Element of Sensor Design. ACS Sens. 2019, 4 (5), 1120–1125. 10.1021/acssensors.9b00260. 31008585

