# Supplementary material for: A Multiplex “Disposable Photonics” Biosensor Platform and Its Application to Antibody Profiling in Upper Respiratory Disease
Source: ACS Sens. 2024 Mar 29;9(4):1799–808. doi: 10.1021/acssensors.3c02225 (PMC11059096; doi:10.1021/acssensors.3c02225)
Supplement: Supplementary file 1 — se3c02225_si_001.pdf [file se3c02225_si_001.pdf]

A Multiplex "Disposable Photonics" Biosensor Platform and its Application to Antibody Profiling in Upper Respiratory Disease

Michael R. Bryan,[1,2‡] Jordan N. Butt,[3‡] Zhong Ding,[8] Natalya Tokranova,[4] Nathaniel Cady,[4] Brian Piorek,[5] Carl Meinhart,[5] Joshua Tice,[6] Benjamin L. Miller[1,2,7,*]

[1]Department of Dermatology, University of Rochester, Rochester, NY 14627

[2]Department of Biochemistry and Biophysics, University of Rochester, Rochester, NY 14627

[3]Department of Chemistry, University of Rochester, Rochester, NY 14627

[4]Department of Nanoscale Science & Engineering, University at Albany, Albany NY 12203

[5]University of California at Santa Barbara, Santa Barbara, CA 93106

[6]QuidelOrtho, Inc., Rochester, NY 14626

[7]The Institute of Optics, University of Rochester, Rochester, NY 14627

[8]ZDing Tech, LLC, Pittsford, NY 14534

[‡]These authors contributed equally

*Author to whom correspondence should be addressed: benjamin_miller@urmc.rochester.edu

Supplementary information

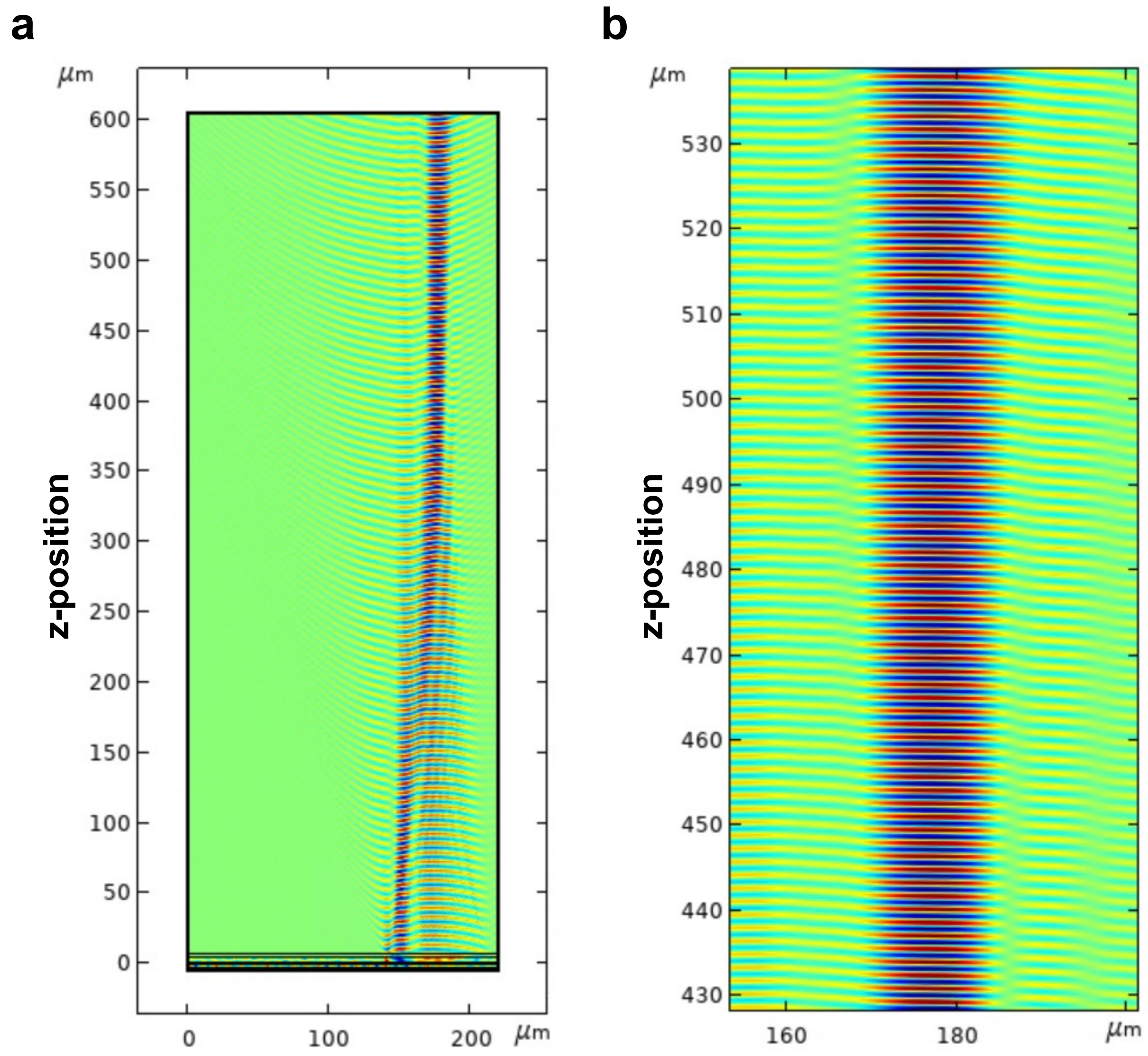

Supplementary Figure S1: Magnitude of the out-of-plane electric field for the grating coupler focused at f_z = 500 μm: (a) entire computational domain, (b) closeup of the focus region.

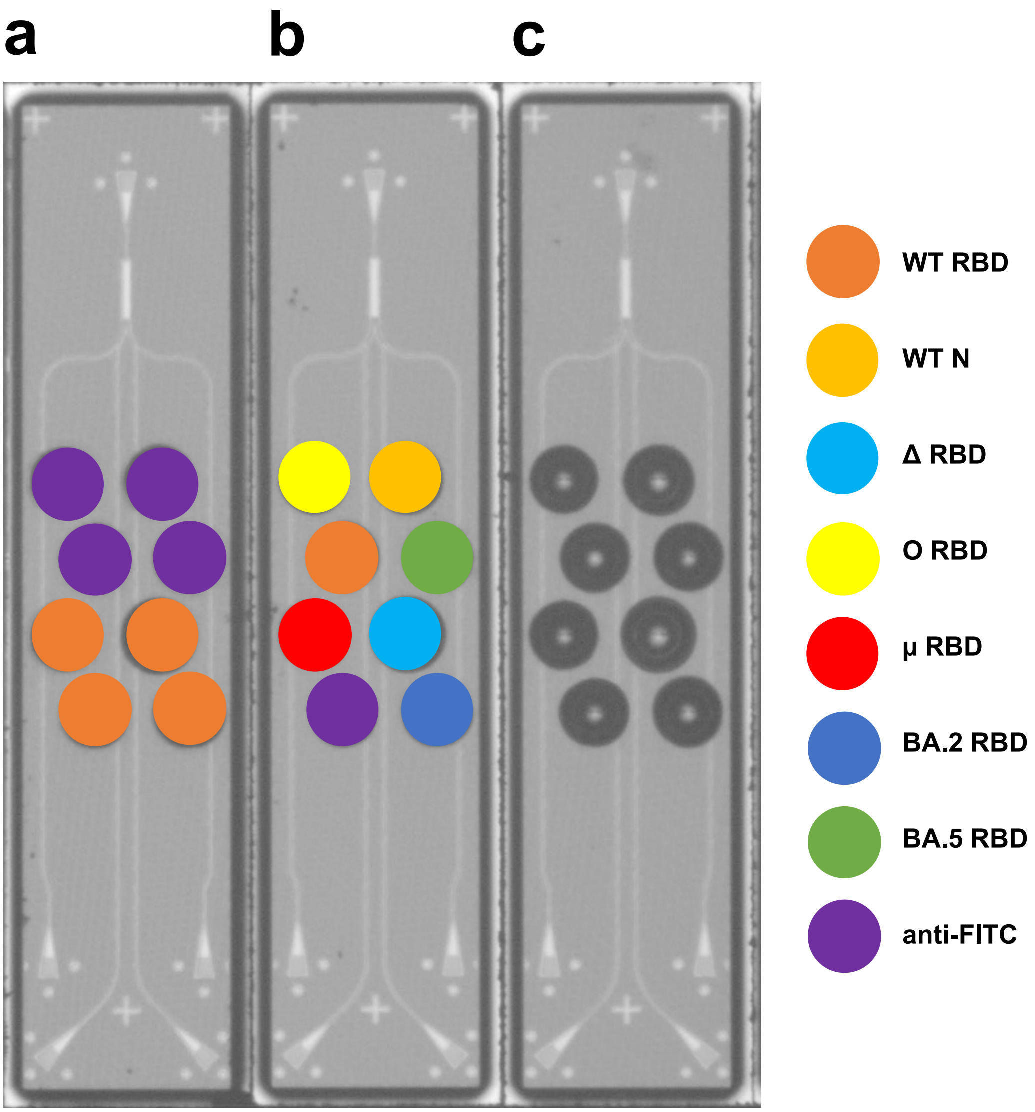

Supplementary Figure S2: Representative functionalization of multiplex 'disposable photonics' PICs. Other variations were also tested in the context of this research. (a) Functionalization for 'singleplex' experiment with replicates. Four rings were printed with anti-FITC as a negative control and four rings are printed with RBD antigen. In principle, one could use a human anti-IgG as a positive control. In practice, we have not found that to be necessary; changes in bulk refractive index once the diluted serum sample is added are sufficient to provide confidence the sensor is working. (b) Functionalization for an 8-plex (7 experimental plus 1 control) experiment with anti-FITC (control), wild-type RBD (WT RBD), wild-type nucleocapsid protein (WT N), Δ

RBD, O RBD, μ RBD, BA.2 RBD, and BA.5 RBD antigens printed on one ring each. (c) Image of a representative PIC after printing capture probes and overspotting with StabilGuard, showing fidelity of the printing process.

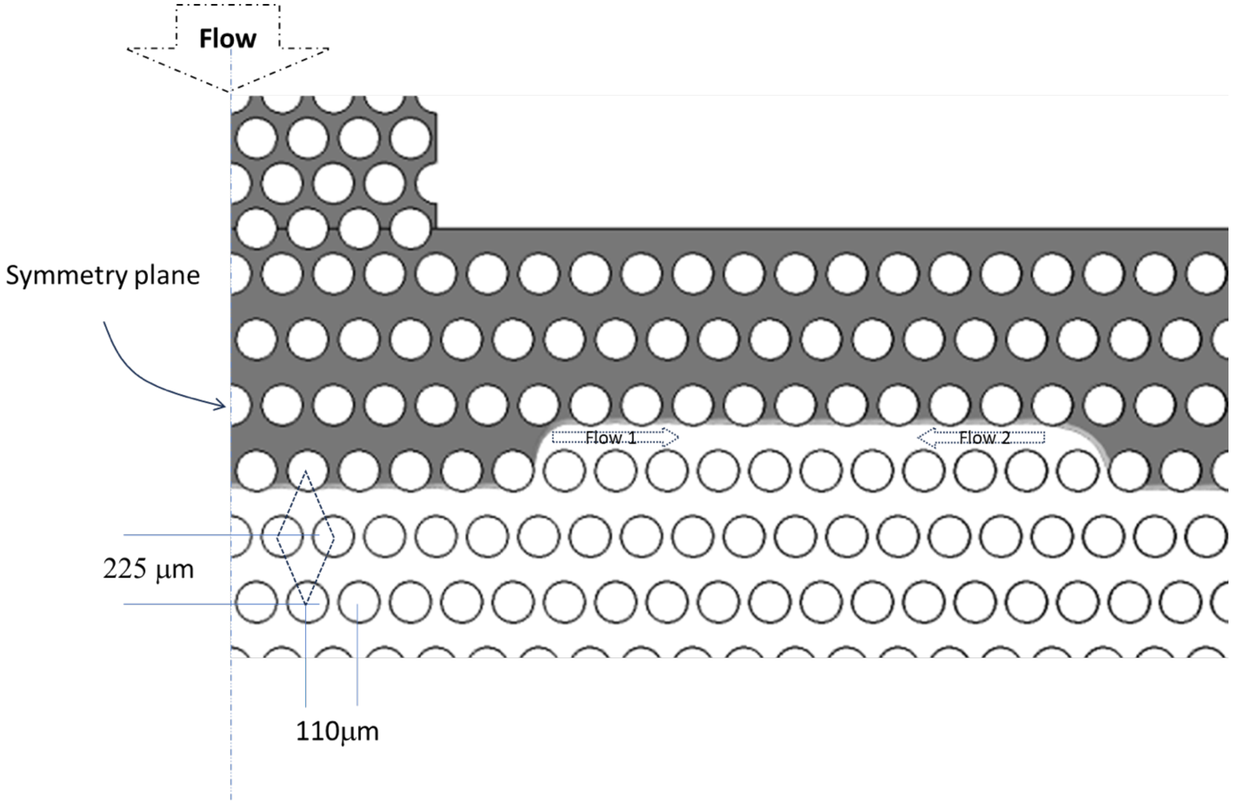

Supplementary Figure S3: COMSOL model showing top view of flow in the in the wicking zone driven by capillary pressure. Fluid fills between each row (Flow 1 and Flow 2 in the figure) before advancing to the next row.

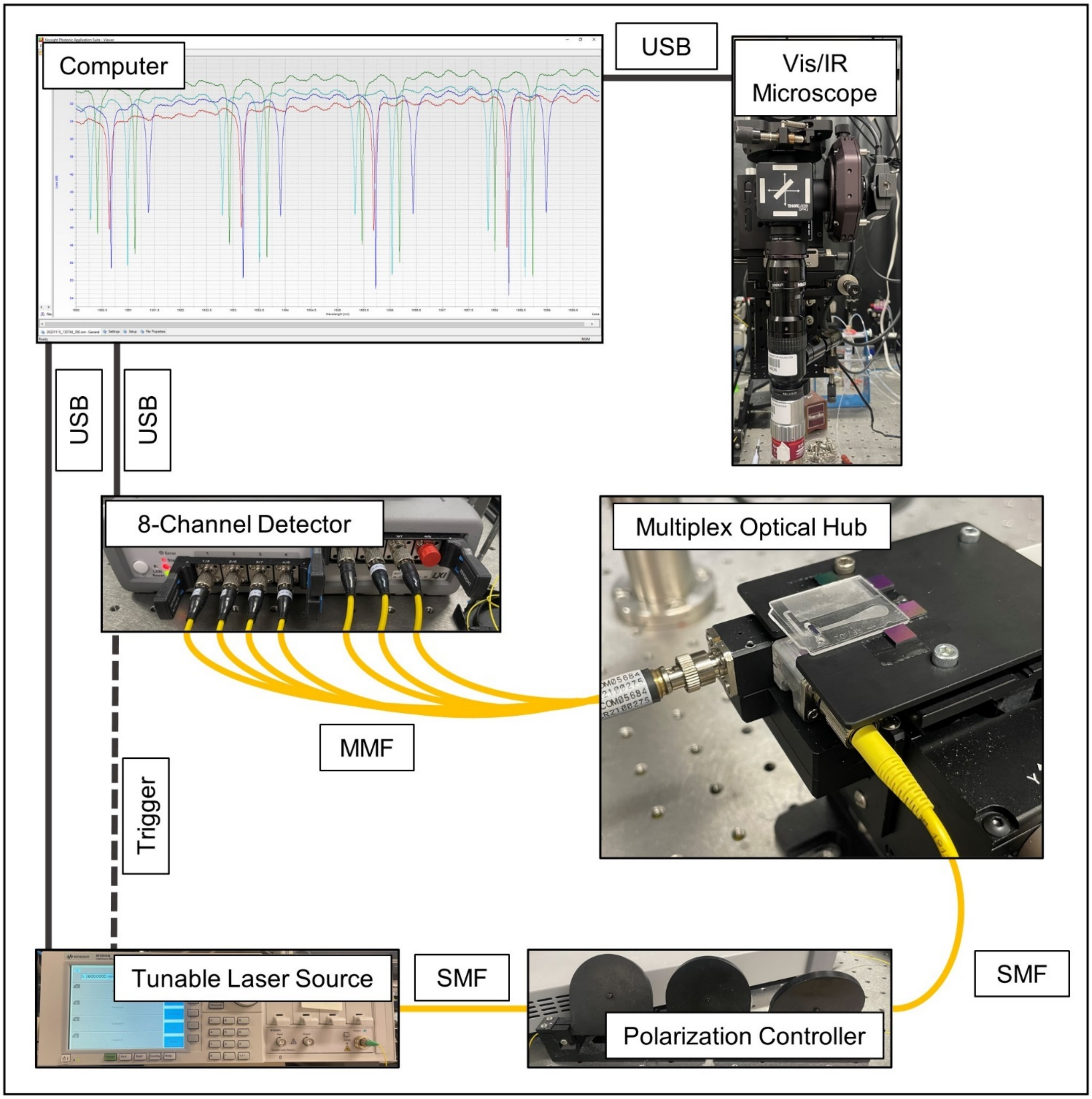

Supplementary Figure S4: Schematic representation of the multiplex optical hub photonic biosensing apparatus. Vis, visible-wavelength light; IR, infrared-wavelength light; SMF, single-mode fiber; MMF, muti-mode fiber; USB, universal serial bus.

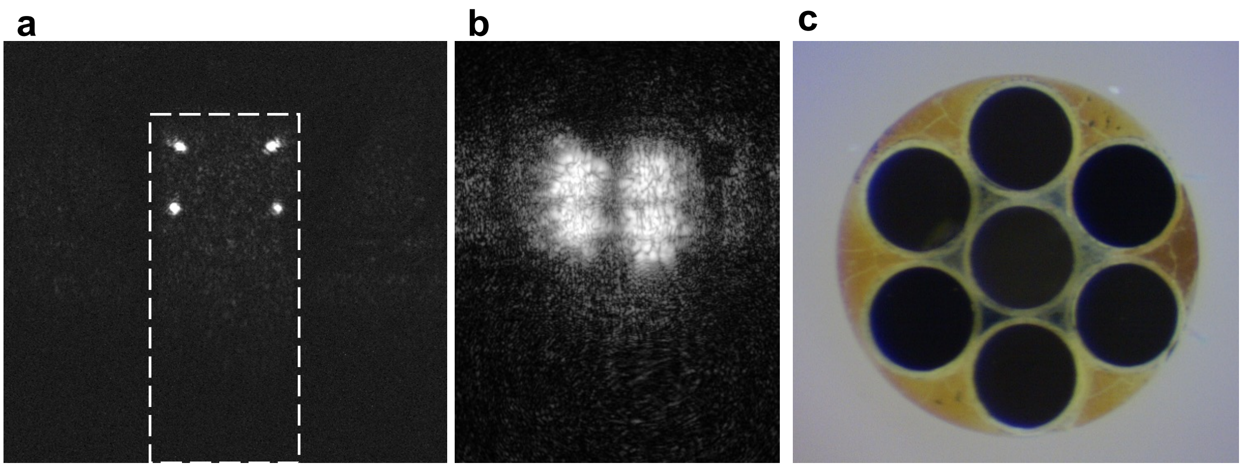

Supplementary Figure S5: Alignment of fiber bundle to output gratings of multiplex PIC

(a) IR micrograph of light emitted from output gratings of a multiplex PIC with the input grating aligned to the hub. Dashed line indicates the perimeter of the PIC. (b) Light routed in reverse through the output multimode fibers of the fiber bundle reveals their alignment relative to the PIC (black outline). c) End view of a fiber bundle used in this work.

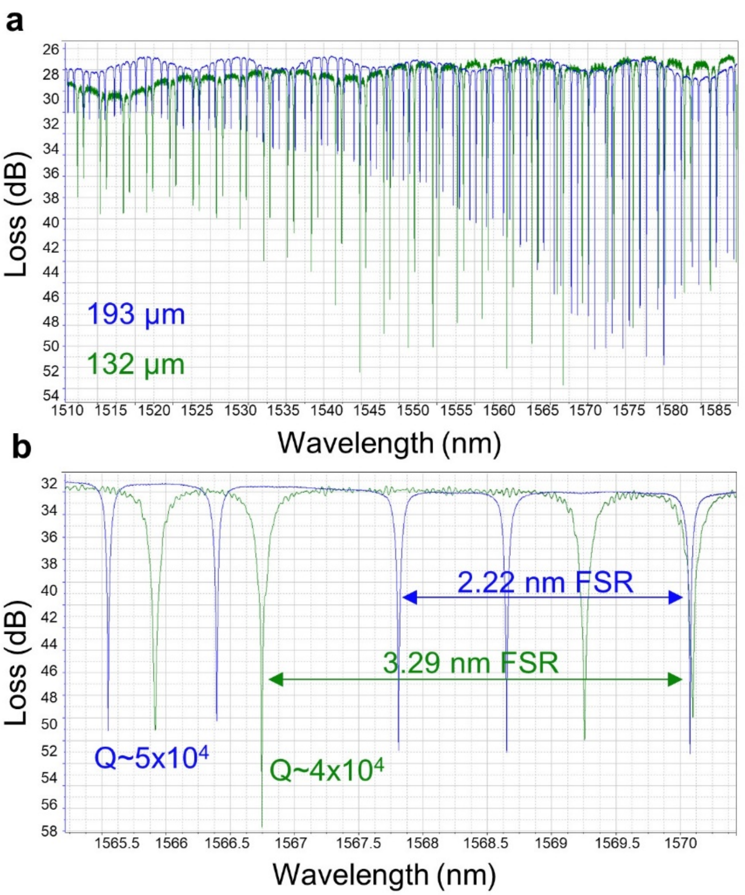

Supplementary Figure S6: (a) Spectra for ring resonators with 193 µm (blue) and 132 µm (green) diameters. The ring with smaller diameter maintains a greater extinction ratio over a broader range of wavelengths. **(b)** Increased bending losses resulting from the smaller diameter ring result in a modest decrease in quality factor (Q), and a significant increase in free spectral range (FSR).

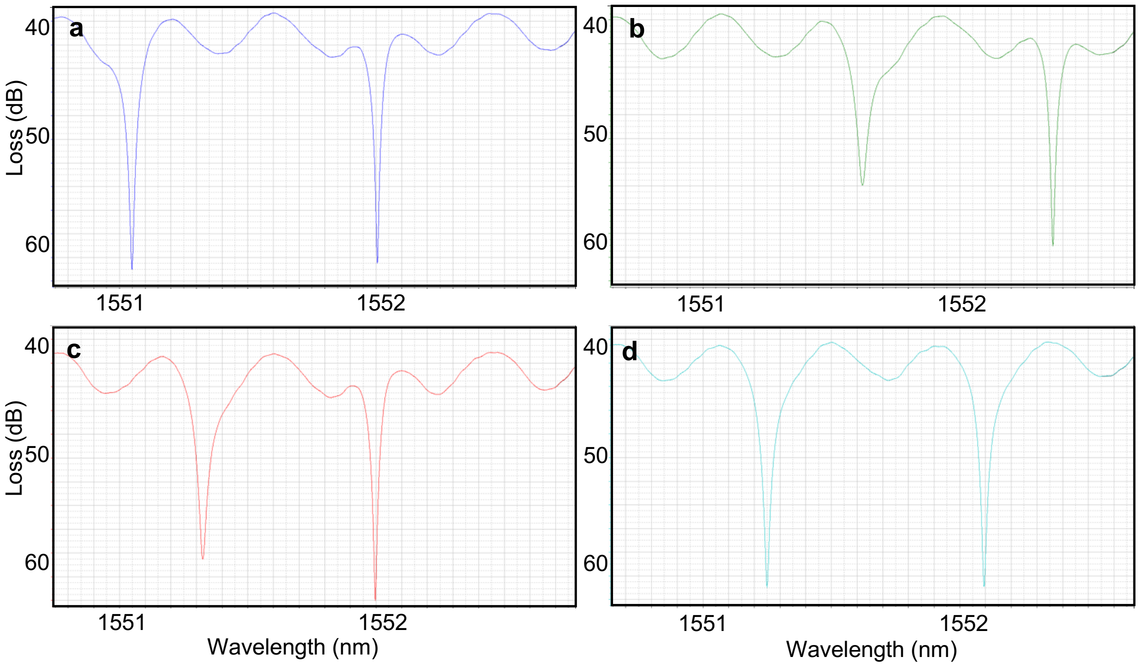

Supplementary Figure S7: Individual spectra from a single PIC highlight a lack of cross-talk between channels.

	Diameter (µm)	FSR (nm)	Q-Factor
Singleplex	198	2.14	4.9E+04
Multiplex	162	2.54	4.5E+04

Supplementary Table S1: Comparison of fabricated rings on Singleplex vs. Multiplex PICs.

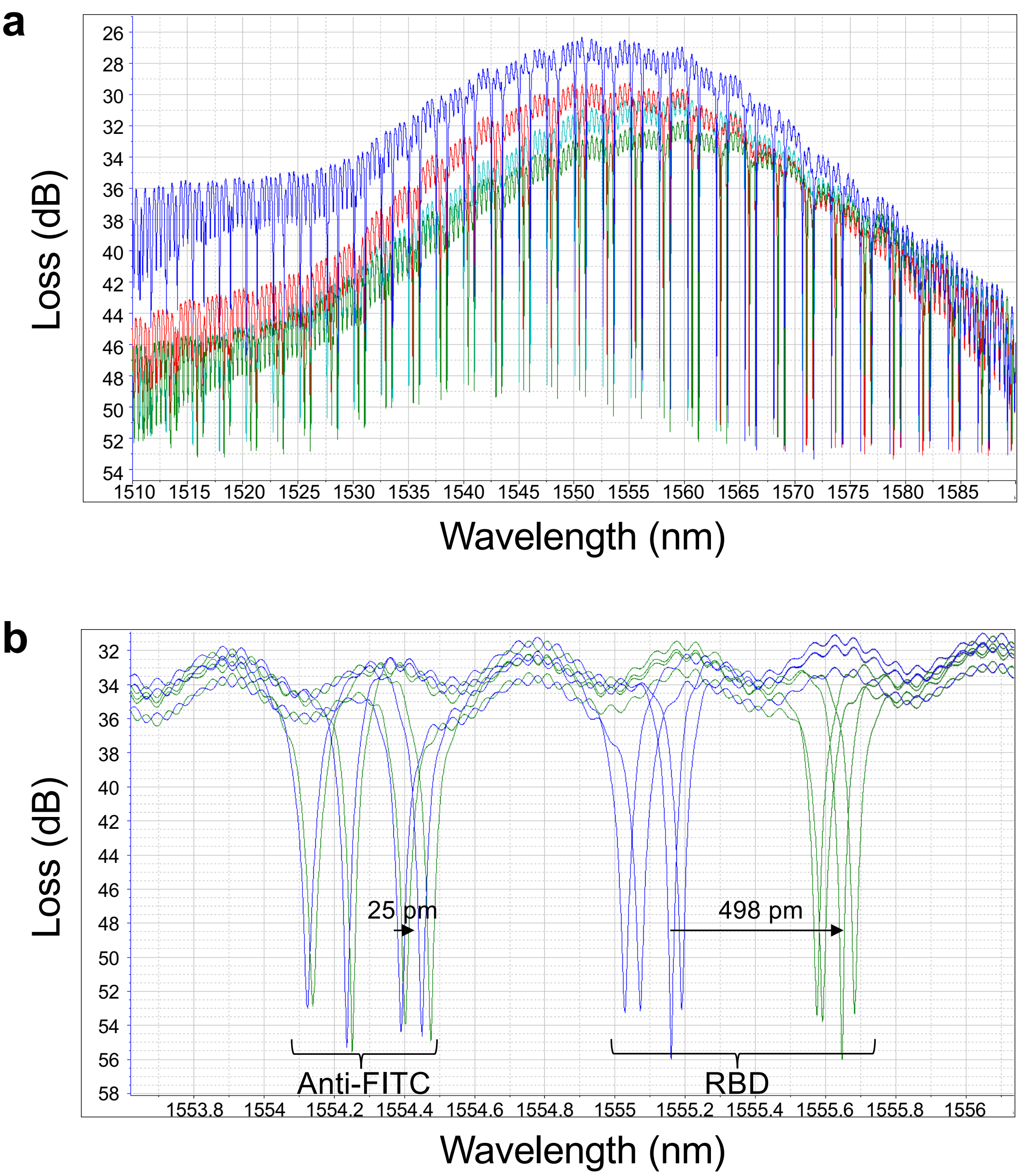

Supplementary Figure S8: (a) Example resonance shift time course. **(b)** Representative spectra from a sample assay showing initial spectra (blue) and 10 minutes after sample addition (green), showing small nonspecific binding shifts for the control protein (Anti-FITC) and more substantial, specific red shifts for the antigen of interest (RBD). Only representative shifts are shown; those from other anti-FITC / RBD rings are similar.

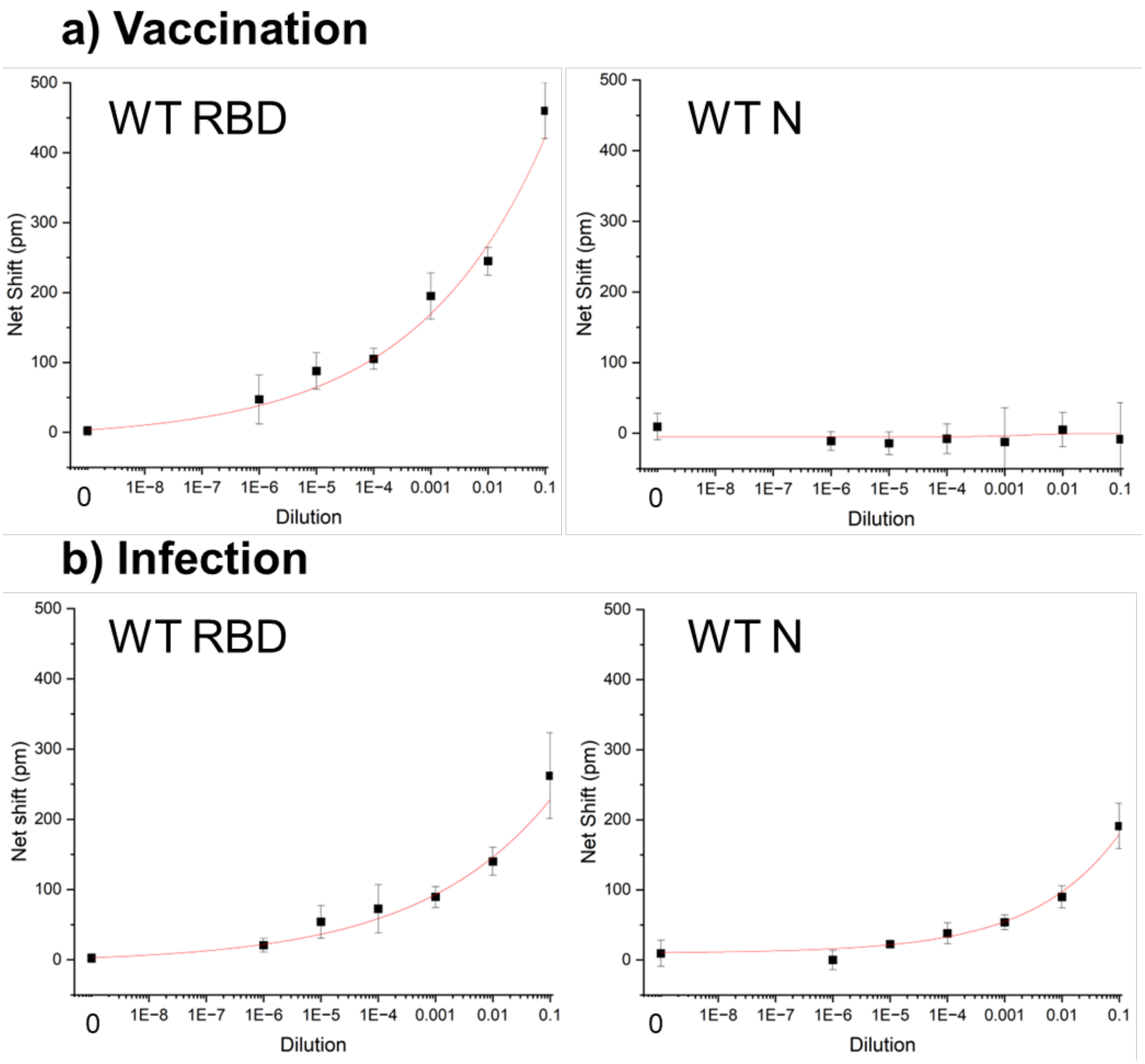

Supplementary Figure S9: Representative serial dilution curves showing response of pooled human sera following vaccination (a) or infection (b). These curves along with response data for δ, μ, ο, BA.2, and BA5 RBD obtained as part of an 8-plex experiment were used to determine detection limits shown in Table S1.

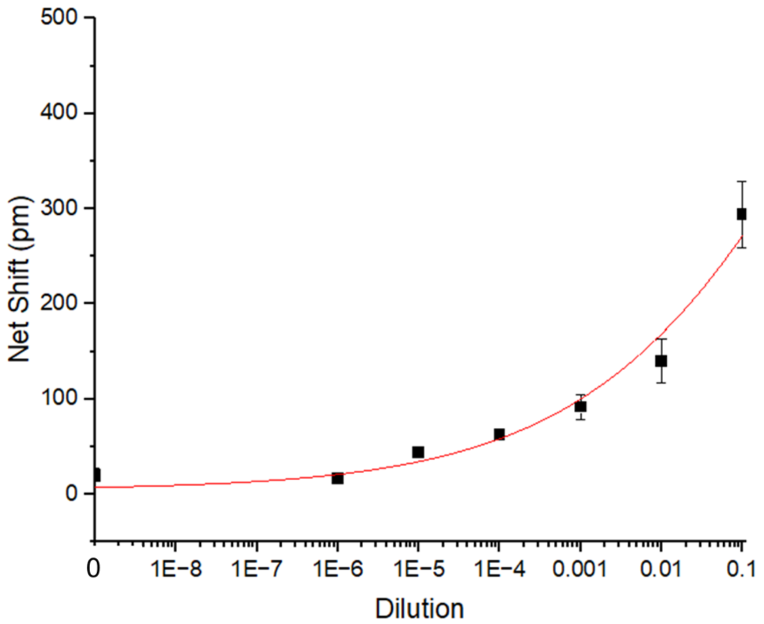

Supplementary Figure S10: Serial dilution of the pooled vaccination sample run on "singleplex" sensors for WT RBD (anti-FITC as negative control).

Protein	LLOD (pooled vaccinated)	LLOD (pooled post-COVID)
WT RBD	106482	418513
WT N	N/A	531
δ RBD	99826	2908
μ RBD	207	888
o RBD	12700	4709
BA.2 RBD	2080	436
BA.5 RBD	1518	213
WT RBD (Singleplex)	111760	ND

Supplementary Table S2: Limits of detection for multiplex analysis pooled human serum samples (serial dilutions, expressed as unitless ratios 1:x)

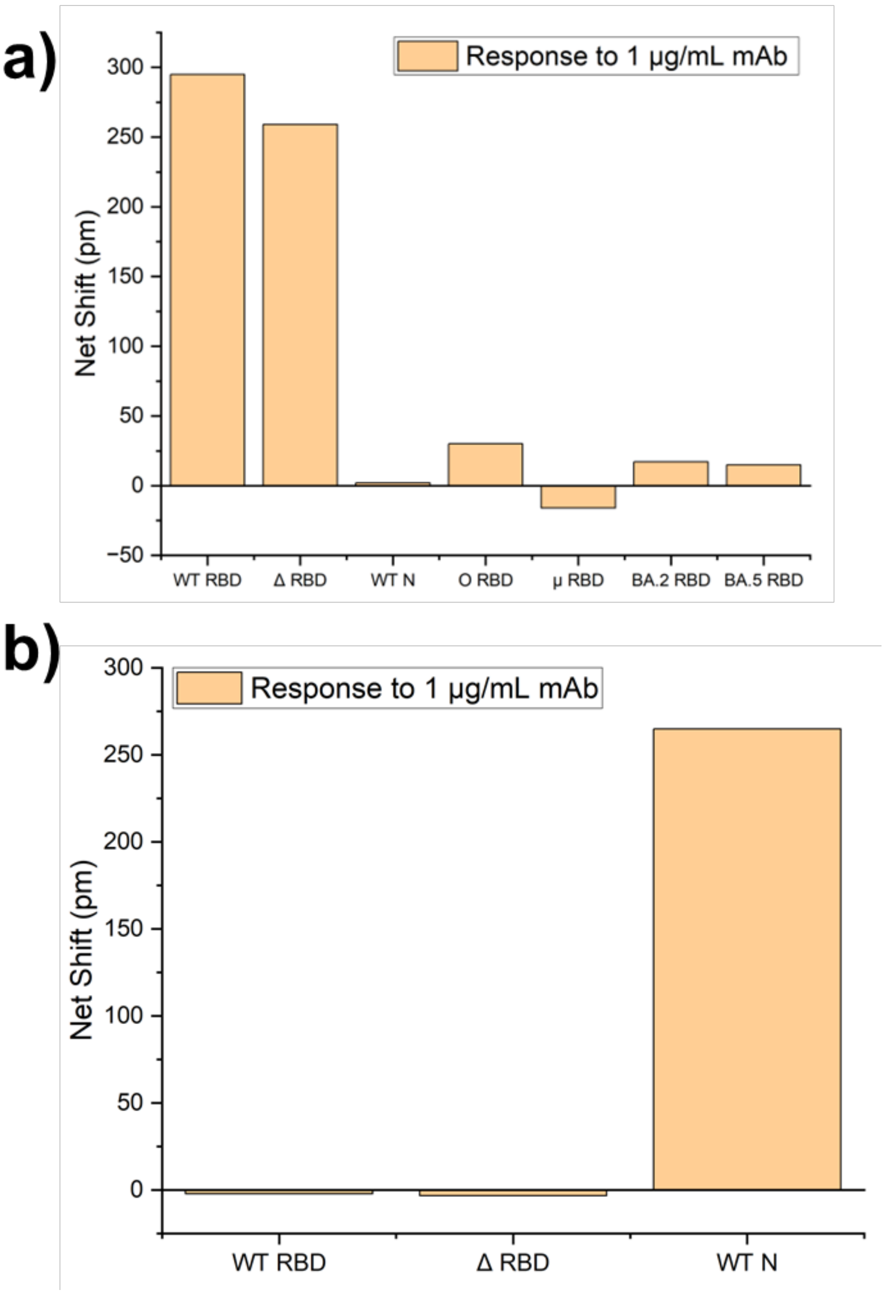

Figure S11: Using the detection platform using monoclonal antibodies to (a) WT RBD and (B) WT N at 1 µg/mL concentrations.

Supplementary Table S3: Cost of Goods Sold (COGS) estimate for the consumable

COMPONENT	COST
1x4 mm PIC	$0.85
Micropillar Card	$1.00
Antigens	$0.12
Packaging	$0.05
Manufacturing, etc.	$0.03
**Total COGS**	**$2.05**
